# L-Citrulline Supplementation Reduces Blood Pressure and Myocardial Infarct Size under Chronic Intermittent Hypoxia, a Major Feature of Sleep Apnea Syndrome

**DOI:** 10.3390/antiox11122326

**Published:** 2022-11-24

**Authors:** Bilgehan Ozcan, Britanny Blachot-Minassian, Stéphanie Paradis, Lucile Mazière, Marie Chambion-Diaz, Sophie Bouyon, Jean-Louis Pépin, Vincent Pialoux, Claire Arnaud, Christophe Moinard, Elise Belaidi

**Affiliations:** 1 Univ. Grenoble Alpes, HP2, Faculté de Pharmacie, F-38042 Grenoble, France; 2 INSERM, U1300, F-38042 Grenoble, France; 3 Université Grenoble-Alpes, Laboratoire de Bioénergétique Fondamentale et Appliquée, INSERM U 1055, Faculté de Pharmacie, 38000 Grenoble, France; 4 Univ. Lyon, Université Claude Bernard Lyon1, Laboratoire Interuniversitaire de Biologie de la motricité, Team ATPA, Faculté de médecine Rockfeller, 69000 Lyon, France; 5 Centre Hospitalier Universitaire Grenoble-Alpes, F-38042 Grenoble, France

**Keywords:** obstructive sleep apnea, citrulline, intermittent hypoxia, oxidative stress

## Abstract

Intermittent hypoxia (IH) is a landmark of obstructive sleep apnea (OSA) at the core of the cardiovascular consequences of OSA. IH triggers oxidative stress, a major underlying mechanism for elevated blood pressure (BP) and increased infarct size. L-citrulline is an amino acid that has been demonstrated to be protective of the cardiovascular system and exert pleiotropic effects. Therefore, we tested the impact of citrulline supplementation on IH-induced increase in BP and infarct size. Four groups of rats exposed to normoxia (N) or IH [14 days (d), 8 h/day, 30 s-O_2_ 21%/30 s-O_2_ 5%] and were supplemented or not with citrulline (1 g·kg^−1^·d^−1^). After 14 d, BP was measured, and hearts were submitted to global ischemia-reperfusion to measure infarct size. Histological and biochemical analyses were conducted on hearts and aorta to assess oxidative stress. Citrulline significantly reduced BP (–9.92%) and infarct size (–18.22%) under IH only. In the aorta, citrulline supplementation significantly decreased superoxide anion and nitrotyrosine levels under IH and abolished the IH-induced decrease in nitrite. Citrulline supplementation significantly decreased myocardial superoxide anion levels and xanthine oxidase enzyme activity under IH. Citrulline shows a cardioprotective capacity by limiting IH-induced pro-oxidant activity. Our results suggest that citrulline might represent a new pharmacological strategy in OSA patients with high cardiovascular risk.

## 1. Introduction

Obstructive sleep apnea (OSA) is a breathing disorder occurring during sleep that affects 1 billion people worldwide [1]. OSA is known to be an independent risk factor for cardiovascular diseases as it increases the risk of atherosclerosis, hypertension, stroke [2], coronary heart disease [3], and myocardial infarction [4]. Several metanalyses are consistent in demonstrating a dose-response relationship between blood pressure (BP) and sleep apnea severity [5]. It was demonstrated that OSA patients exhibit larger infarct sizes and lower left ventricular ejection fraction at 3 months after an acute myocardial infarction compared to non-apneic patients [6].

Continuous positive airway pressure (CPAP) is the first line of therapy for OSA, applying air pressure to keep the airways open during sleep. CPAP therapy is highly efficient in improving quality of life and excessive daytime sleepiness, but its impact is still debated regarding the reduction in incident cardiovascular events. A recent randomized controlled trial demonstrated that CPAP treatment does not prevent cardiovascular events [7]. These results are, however, challenged by real-world evidence in clinical cohorts reporting more positive results [8,9]. Another major issue with CPAP is long-term adherence to therapy. In a study including a total of 480,000 patients, the CPAP termination rate after 3 years was 47.7%, demonstrating the need for alternative treatments [10,11,12]. In addition, OSA is associated with several co-morbidities, such as resistant hypertension [13] or obesity [14,15], requiring combined therapies of lifestyle interventions [16] and new pharmacological agents. Therefore, it is paramount to better understand the mechanisms underlying OSA-related cardiovascular diseases to propose new pharmacological targets to be used as the primary therapy after a CPAP refusal or in combination with CPAP and/or lifestyle interventions.

OSA is characterized by repetitive upper airway obstructions leading to intermittent hypoxia (IH), the major trigger of OSA cardiovascular consequences [17]. In preclinical models of mice and rats exposed to IH, IH increased mean blood pressure (MBP) [18,19] as well as infarct size upon ischemia-reperfusion in rats [20] and mice [21]. Among the mechanisms involved, hypoxia-inducible factor 1 (HIF-1) plays a central role [22]. IH is a potent inducer of oxidative stress [23,24] that strongly activates HIF-1, which in turn exacerbates oxidative stress by increasing the expression and activity of NADPH oxidase 2 [25], and superoxide anion (i.e., O_2_^.−^) production [26]. Under chronic IH, the suppression of oxidative stress by tempol or atorvastatin prevents the increase in infarct size and elevated blood pressure [18,27]. The inhibition of HIF-1 activation by curcumin decreased superoxide anion production [28] and reduced myocardial injury. Despite this strong mechanistic rationale, the impact of anti-oxidant treatment in OSA has not been investigated in large clinical trials [22].

In endothelial cells, nitric oxide (NO) is synthesized by endothelial nitric oxide synthases (eNOS) using arginine in the presence of NADPH, oxygen, and calcium. NO production has been consistently described as being impaired by oxidative stress through direct NO interaction with superoxide to form peroxynitrite (ONOO^−^) and by limiting eNOS [29]. Chronic IH significantly decreased eNOS expression and increased arginase expression in carotid arteries of rats [30], decreasing NO bioavailability and resulting in endothelial dysfunction [31,32,33]. Moreover, it was also reported that the OSA patients had increased plasmatic levels of asymmetrically dimethylated arginine (ADMA), which is an eNOS inhibitor involved in cardiovascular diseases [33].

L-citrulline is a non-proteinogenic and non-essential amino acid, best known to be the precursor of L-arginine. Citrulline supplementation has been demonstrated to be much more efficient in increasing plasmatic arginine concentration than arginine supplementation itself [34]. In humans and rodents, citrulline supplementation has shown beneficial effects on the cardiovascular system. In spontaneously hypertensive rats, citrulline supplementation was reported to decrease BP, supposedly by restoring NO bioavailability [35]. In rabbits under a high-cholesterol diet, citrulline preserved endothelial function by decreasing superoxide anion production [36]. Citrulline also improved the cardiac function of isolated and perfused rat hearts [37] and decreased infarct size in db/db mice, a model of diabetic and obese mice [38]. Furthermore, citrulline supplementation is known to be safe in humans [39]. Clinical trials demonstrated that citrulline supplementation decreased BP in healthy overweight/obese men [40] and improved ejection fraction in patients with heart failure [41].

We hypothesized that citrulline could protect against IH-induced elevated BP and an increase in myocardial infarct size. The aim of this study was to assess, in rats, the impact of citrulline supplementation during IH on (1) BP and infarct size in response to myocardial ischemia-reperfusion; (2) aortic and myocardial oxidative stress.

## 2. Materials and Methods

### 2.1. Ethical Approval

The experimental procedures were conducted in accordance with the European Convention for the Protection of Vertebrate Animals used for Experimental and Other Scientific Purposes (Council of Europe, European Treaties ETS 123, Strasbourg, 18 March 1986) and with the Guide for Care and Use of Laboratory Animals (NIH Publication No. 85-23, revised 1996) and were approved by an Institutional Animal Care and Use Committee (agreement number 201603301129626-V3#7130).

### 2.2. Animals and Exposition to Normoxia or Intermittent Hypoxia

Seventy-nine Wistar male rats (6-week-old, 270–350 g, Janvier Labs, Le Genest-Saint-Isle, France) were housed at the animal care facility of the HP2 Laboratory, under a 12:12 hour light-dark cycle at 20–22 °C and allowed free access to standard food and water during one week of acclimatization. They were divided into two sets (set 1 and set 2, *n* = 56 and *n* = 23, respectively). Based on our experimental knowledge and our conditioning constraints, each set of rats was randomized into 4 groups (*n* = 14 and *n* = 5–6, set 1 and 2, respectively): Normoxia (N), intermittent hypoxia (IH) and citrulline supplemented (1 g·kg^−1^·d^−1^ in food), normoxia (NCit) or intermittent hypoxia (IHCit)) submitted to IH [14 days (d), 8 h·d^−1^, 30 s-O_2_ 21%/30 s-O_2_ 5%], or N [14 d, 8 h·d^−1^ 30 s-O_2_ 21%/30 s-O_2_ 21%]. As previously described [42], control animals in the N groups were exposed to similar 1-min air-air cycles to reproduce noise, air turbulence, and stress similar to the IH stimulus. 

During the procedure, rats and food were weighed daily in order to adapt the production of citrulline-supplemented food (Citrulline was a gift from Citrage company). The control groups were exposed to N or IH one week before starting citrulline supplementation in order to adjust the citrulline levels in the food.

**Figure 1:**
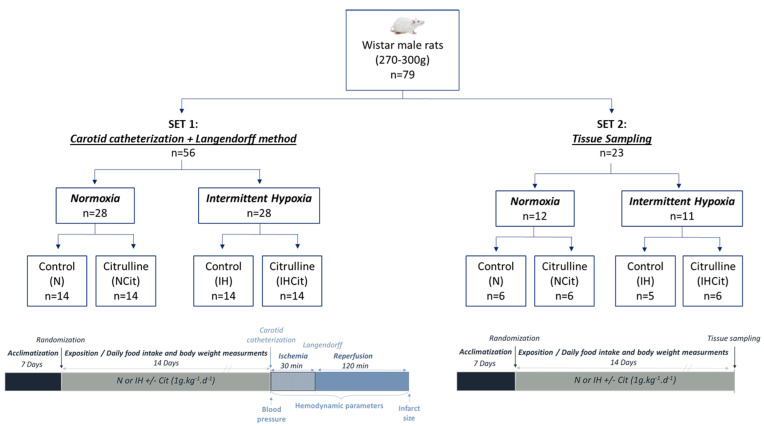
Experimental groups and design. Seventy-nine rats were separated into 2 sets. Both sets of rats were randomized into normoxia (N) or intermittent hypoxia (IH; 8 h per day, 30 s of 21% O_2_° and 30 s of 5% O_2_) for 14 days, supplemented with 1 g·kg^−1^·d^−1^ citrulline (Cit) or not (Control). At the end of exposure, rats from set 1 were subjected to blood pressure measurements by carotid catheterization. Afterward, the hearts were isolated and perfused by the Langendorff method and submitted to ischemia (30 min) and reperfusion (120 min) protocols, during which the hemodynamic parameters and, in the end, infarct size were measured. Rats from set 2 were euthanatized after 14 days of exposure for blood collection and tissue sampling for further biochemical analyses.

The first set of rats was used to measure BP and infarct size. The second set was used for tissue sampling to explore oxidative stress (Figure 1).

### 2.3. Carotid Catheterization, Langendorff, and Ischemia-Reperfusion

Rats were anesthetized with an intraperitoneal injection of sodium pentobarbital (60 mg·kg^−1^) and maintained on a heated pad (37 °C) during the whole experiment. After an intravenous injection of 950 IU·kg^−1^ of heparin, the neck of the rat was opened in order to isolate the left carotid artery. A distal ligature was placed on the rostral side while the caudal side of the carotid was clamped. Then, a heparinized catheter, linked to a mecano-transducer, was inserted into the carotid artery, and systolic (SBP), diastolic (DBP), and MBP were recorded and measured for 1 min using PowerLab 26 T (ADInstruments). 

A thoracotomy was performed, and the heart was removed and placed in a 4 °C Krebs-Henseleit solution (NaCl, 119 mM; glucose, 11 mM; NaHCO_3_, 22 mM; KCl, 4.7 mM; MgCl_2_, 1.2 mM; KH_2_PO_4_, 1.2 mM; CaCl_2_, 2.5 mM; EDTA, 0.5 mM; pyruvate, 2 mM) [42]. The aorta was cannulated on a gauge needle and perfused immediately with Krebs-Henseleit solution bubbled with a mix of 95% O_2_ and 5% CO_2_ at 37 °C and at a constant pressure of 80 mmHg. A thermometer was inserted into the left ventricle in order to control the temperature. A polyvinyl chloride balloon filled with water was also introduced into the left ventricle through the left atrium. The balloon was connected to a pressure transducer (ADInstruments) and inflated to obtain the left ventricular end-diastolic pressure (LVEDP) between 5 and 15 mmHg. After that, the left ventricle developed pressure (LVDP), left ventricular derivatives of pressure over time at its maximum and minimum (dp/dt max and min), coronary flow (CF), and heart rate (HR) were measured.

After 20 min of stabilization, the hearts were submitted to 30 min-global and total ischemia by stopping the perfusion and 120 min-reperfusion. In the end, the hearts were collected and cut into 6 transversal slices. The slices were incubated with 1% triphenyl tetrazolium chloride (TTC, Sigma-Aldrich, Saint-Quentin Fallavier, France) in a phosphate buffer (42 mM KH_2_PO_4_, 131.2 mM K_2_HPO_4_ at pH = 7.4) and fixed with 4% of formaldehyde overnight. Infarct size was determined by a planimetric technique, analyzed using ImageJ (NIH), and expressed relative to the total area of the left ventricle.

Exclusion criteria: BP data measured with a technical problem (carotid perforation, too much time to measure BP (*n* = 3 N, *n* = 3 NCit, *n* = 2 IHCit)) or from rats with a heart rate not ranged between 300 and 500 beats·min^−1^ (*n* = 1 IH, *n* = 2 IHCit) were excluded. The infarct size data from hearts with a Langendorff perfusion problem (*n* = 3 N, *n* = 2 NCit, *n* = 1 IHCit) or with arrhythmia (*n* = 1 N, *n* = 1 NCit) during the stabilization period were excluded.

### 2.4. Plasma, Heart, and Aorta Sampling and Extraction

Rats of the second set were anesthetized with an intraperitoneal injection of sodium pentobarbital (60 mg·kg^−1^). After a thoracotomy, blood was collected with intraventricular punctuation in EDTA-coated tubes (vacuum) and centrifuged at 4 °C, 4000 rpm for 10 min. The plasmas were collected and preserved at −80 °C. Blood was also collected in microhematocrit capillary tubes (HIRSCHMANN NA-Heparin) and centrifuged (13,000 rpm for 7 min at 21 °C) to measure hematocrit. The heart and aorta were collected, and a slice was cryopreserved in Shadon Cryomatrix (Thermo Scientific) embedding resin, while the rest was frozen in liquid nitrogen and preserved at −80 °C.

Whole protein extraction was performed as previously described [43]. Protein concentration was measured using a Bradford assay (Sigma-Aldrich, St. Louis, MO, USA).

Exclusion criteria: Due to the high variability of the collected aorta size, aorta protein extracts presenting less than 20 mg·mL^−1^ of protein (*n* = 2 N, *n* = 1 IH) were excluded from further biochemical analyses.

### 2.5. Oxidative Stress Markers

#### 2.5.1. Dihydroethidium (DHE)

The cryoconcerved sections of the heart and aorta in embedding resin were cryosectioned (10 µm thick) at −20 °C (Cryostat Leica CM1950) and collected on Superfrost Plus slides (Deutscher, France). The slides were dried for 10 min, stained with 4′,6-diamidino-2-phenylindole (DAPI) 25 µM for 10 min, and with DHE 2.5 µM (Sigma-Aldrich) for 5 min. While DAPI binds with ADN and yields blue fluorescence, DHE reacts with superoxide anion in cells and produces ethidium bromide that intercalates in DNA and generates red fluorescence upon excitation. The fluorescence was studied using Zeiss, Axioplan2 imaging microscopy (equipped with an eyepiece PI 10×/25), ×10 objective was used, and the images were analyzed with ImageJ 1.53k software. The calculated percentages of fluorescence were reported as fold increases compared to the mean of the N group.

#### 2.5.2. Advanced Oxidation Protein Products (AOPP)

Protein extracts from hearts were mixed with PBS and acetic acid 98–99%. In parallel, the concentration standards were prepared with chloramine-T (0–0.2 mM), potassium iodide at 1.16 M, and acetic acid at 98–99% in 96 well plates [44]. The absorbance was measured at 340 nm by spectrophotometry and expressed in mmol·mg^−1^ of proteins.

#### 2.5.3. Pro- and Anti-Oxidant Enzyme Activities

Superoxide dismutase (SOD) activity in heart and aorta protein extracts was determined using a method described in [45]. Briefly, 250 µL of a cocktail solution (50 mM Tris-HCl, 0.07 mM nitroblue tetrazolium (NTB), 1.07 mM Diethylenetriamine penta-acetic acid (DTPA), 0.19 mM hypoxanthine) were distributed into the wells, with 20 µL of protein extract. In the presence of 20.4 mU of xanthine oxidase in the wells, the absorbance of produced blue formazan was measured at 450 nm for 5 min. The data were expressed in µmol·min^−1^·g^−1^ of proteins.

Catalase activity of heart samples was determined according to the method described in [46] by a formaldehyde standard (0–0.65 mM). Mixed with 20 µL of protein extracts were 100 µL of PBS, 30 µL of methanol (100%), and 20 µL of H_2_O_2_ (0.144%). The reaction was allowed for 20 min and stopped with 30 µL of KOH (10.7 M) in each well, followed by 30 µL of Purpald (0.2 M in 0.5 N HCL). The absorbance of the formaldehyde formed in wells was measured at 540 nm, and the results were expressed as µmol·min^−1^·g^−1^ of proteins.

Glutathione peroxidase (GPX) activity in each sample of heart tissue was measured [47]. A cocktail solution was prepared to contain 4U of Glutathione reductase, 1.7 mM NADPH, and 1.6 mM reduced glutathione. Mixed in wells with 20 µL of protein extracts were 200 µL of PBS, 30 µL of the cocktail solution, and 30 µL of H_2_O_2_ (0.036%). The absorbance was measured at 340 nm for 5 min. Data were reported as µmol·min^−1^·g^−1^ of proteins.

NADPH oxidase (NOX) and xanthine oxidase (XOX) activities were measured in heart and aorta total protein extracts as previously described [48]. Briefly, 250 µL of a cocktail solution (2.2 mM NTB, 2.8 mM Tris-HCl, 1.32 mM DTPA) were distributed in each well, with 20 µL of protein extracts as well as 1 mM of sodium cyanide. For the NOX assay, 100 µM NADPH was added to the wells. The absorbance of the blue formazan produced was measured at 560 nm, and data were expressed as µmol·min^−1^·g^−1^ of proteins. As for the XOX assay, 500 µM hypoxanthine was added to the wells. The absorbance of the blue formazan produced was measured at 560 nm, and data were expressed as µmol·min^−1^·g^−1^ of proteins.

#### 2.5.4. Nitrotyrosine (3-NT)

Nitrotyrosine levels were measured by ELISA via quantification of 3-NT concentration in plasma and aorta protein extracts [49]. In wells coated overnight with a mixture of 0.05 M NaHCO_3_ and 2 µg·mL^−1^ nitrated bovine serum albumin (BSA), the same volume of samples and anti-nitrotyrosine antibody (1:10,000, N0409 Sigma-Aldrich, Saint-Quentin Fallavier, France) were incubated for 90 min. After washing with PBS-Tween (0.05%), the wells were incubated with goat anti-rabbit poly-horseradish peroxidase (HRP) (1:2000 antibody Invitrogen 32260 mixed with HRP conjugate) for 1 h and washed again. Added to the wells was 100 µL of 3,3′,5′-Tétraméthylbenzidine (TMB) solution, and absorbance was read at 650 nm after 20 min of incubation. To stop the reaction, 89.5 mM H_2_SO_4_ solution was added to the wells, and the absorbance was read, this time at 450 nm. Data were expressed as µmol·g^−1^ of aorta proteins and µmol·ml^−1^ of plasma.

#### 2.5.5. Nitrite/Nitrate

NO metabolism end products in plasma and aorta protein extracts were measured by the Griess method. Griess reagent was prepared using 0.06 M sulfanilamide, 5 mM naphthalene-ethylene, diamine dihydrochloride, and 0.5 M phosphoric acid. Samples (20 µL) were directly mixed with 50 µL of water and 20 µL of Griess reagent and then incubated for 5 min for nitrites measurements. For the sum of nitrates, samples were previously incubated for 40 min with 40 µL of a solution containing 83 mU of Nitrate reductase and 66 µM NADPH. In parallel, a concentration standard of sodium nitrate (0–0.1 mM) was prepared, and the absorbance was measured at 550 nm. Data were expressed as µmol·g^−1^.

For all oxidative stress markers (except for DHE), the measurements were done in duplicates. We used products from Sigma-Aldrich, and the absorbance was measured in 96 well plates using the plate reader TECAN Infinite 2000 (Tecan, Männedorf, Switzerland).

### 2.6. Statistical Analyses

The data were presented as the mean ± SD. Statistical significance was calculated using GraphPad’s Prism 9.3.1 software. Outliers were identified by the Rout method at q = 0.5% and excluded from further analyses. A two-way ANOVA test was conducted with IH and citrulline supplementation variables, followed by a post-hoc test of Fisher′s LSD. A *p*-value under 0.05 was considered a significant difference between groups.

Materials and Methods details are described in supplemental materials (Appendix A). 

## 3. Results

### 3.1. Citrulline Has No Effect on Body Weight, Food Intake, and Hematocrit

**Figure 2:**
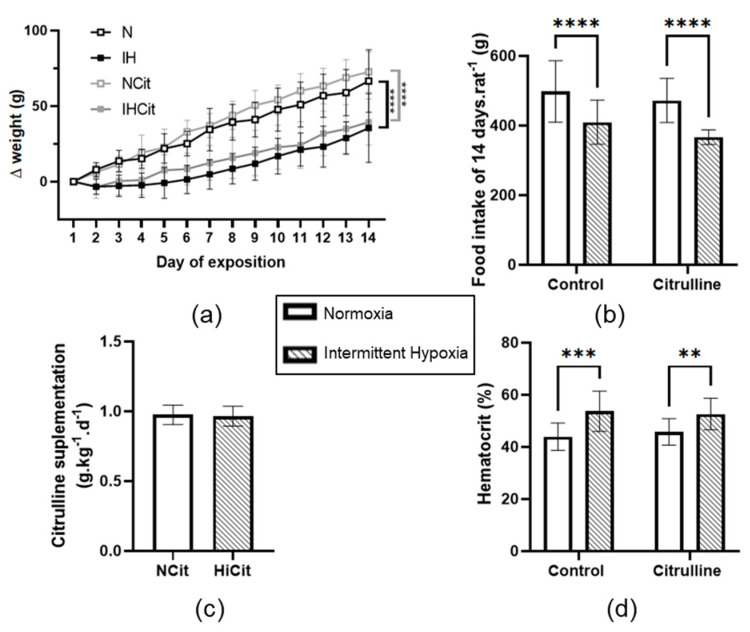
(**a**) Body weight gain, (**b**) food intake, (**c**) mean citrulline supplementation dose (*n* = 19–20), and (**d**) hematocrit (*n* = 13–14) of rats exposed to normoxia (N) or intermittent hypoxia (IH) supplemented with 1 g·kg^−1^·d^−1^ citrulline (Cit) or not (Control). Values are represented as mean ± SD. Two-way ANOVA followed by Fisher’s LSD test. ** *p* < 0.01, *** *p* < 0.001, **** *p* < 0.0001.

Fourteen days of IH exposure decreased body weight gain and food intake compared to N exposure (Figure 2a,b). Citrulline supplementation was calculated and adjusted every day according to the food intake and body weight; N and IH rats ingested a mean of 0.97 ± 0.02 and 0.96 ± 0.02 g·kg^−1^·d^−1^ of citrulline throughout 14 days (Figure 2c). Citrulline supplementation did not prevent an IH-induced decrease in body weight gain and food intake. Furthermore, in both control and citrulline conditions, IH increased significantly hematocrit (Figure 2d).

### 3.2. Citrulline Prevented the IH-Induced Increase in Blood Pressure and Infarct Size

**Figure 3:**
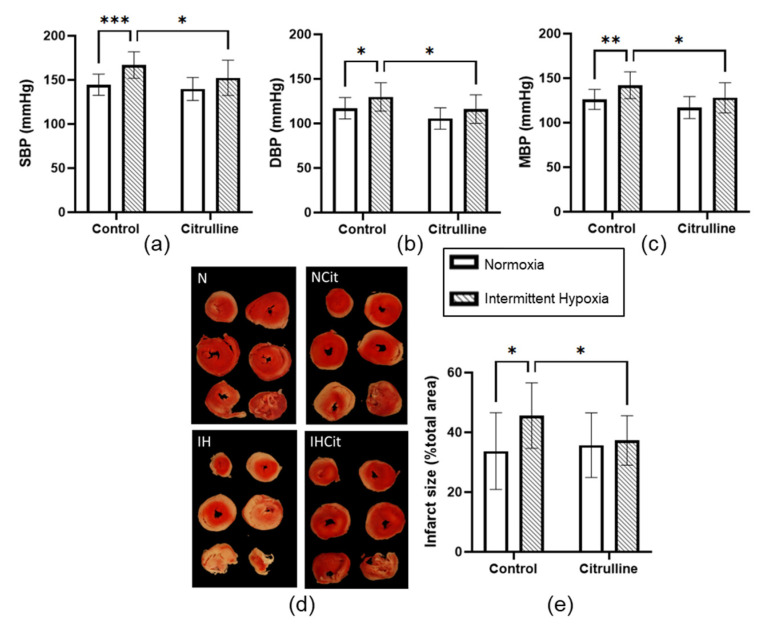
Citrulline supplementation prevented intermittent hypoxia (IH)-induced increase in blood pressure (BP) and infarct size. (**a**) Systolic blood pressure (SBP), (**b**) diastolic blood pressure (DBP), (**c**) mean blood pressure (MBP) expressed in mmHg (*n* = 10–13), (**d**) representative images of triphenyl tetrazolium chloride coloration after the ischemia/reperfusion protocol (red zone: viable area; white zone: infarcted area), (**e**) infarct size expressed relative to total heart area (*n* = 10–14) from rats exposed to normoxia (N) or IH supplemented with 1 g·kg^−1^·d^−1^ citrulline (Citrulline) or not (Control). Values are represented as mean ± SD. Two-way ANOVA followed by Fisher’s LSD test. * *p* < 0.05, ** *p* < 0.01, *** *p* < 0.001.

Fourteen days of IH significantly increased SBP (144.5 ± 3.5 vs. 166.9 ± 4.1 mmHg, in N and IH groups, respectively, *p* < 0.001) (Figure 3a), DBP (117 ± 3.6 vs. 129.8 ± 4.4 mmHg, in N and IH groups, respectively, *p* < 0.05) (Figure 3b), and MBP (126.1 ± 3.4 vs. 142.1 ± 4.2 mmHg, in N and IH groups, respectively, *p* < 0.01) (Figure 3c). An increase in infarct size was observed in IH (33.7 ± 4.4 vs. 45.6 ± 2.9%, in N and IH groups, respectively, *p* < 0.05) (Figure 3d). Citrulline significantly reduced SBP, DBP, MBP, and the infarct size in the IH condition only.

### 3.3. Citrulline Decreased Superoxide Anion Content and Nitrotyrosine Levels in Aorta

**Figure 4:**
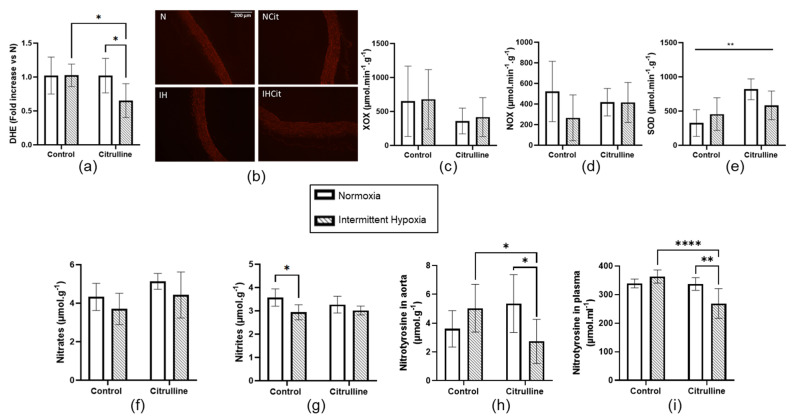
Citrulline decreased oxidative stress, nitric oxide derivatives, and nitrotyrosine levels in the aorta under intermittent hypoxia (IH). (**a**) DHE levels with (**b**) representative images (×100); Activities of (**c**) xanthine oxidase (XOX), (**d**) NADPH oxidase (NOX), and (**e**) superoxide dismutase (SOD) expressed in µmol·min^−1^·g^−1^ of protein (*n* = 5–6); (**f**) nitrate, (**g**) nitrite and (**h**) nitrotyrosine levels in aorta expressed in µmol·g^−1^ (*n* = 4–6) (**i**) Nitrotyrosine levels in plasma expressed in µmol·ml^−1^ (*n* = 5–6) from rats exposed to normoxia (N) or IH supplemented with 1 g·kg^−1^·d^−1^ citrulline (Cit) or not (Control). Values are represented as mean ± SD. Two-way ANOVA followed by Fisher’s LSD test. * *p* < 0.05, ** *p* < 0.01, **** *p* < 0.0001.

IH did not impact DHE levels (Figure 4a,b), nor xanthine oxidase (XOX) (Figure 4c), NADPH oxidase (NOX) (Figure 4d), and superoxide dismutase (SOD) (Figure 4e) enzyme activities in the aorta. Citrulline significantly decreased DHE levels only in the IH group (1.03 ± 0.08 vs. 0.65 ± 0.12, in IH and IHCit groups, respectively, *p* < 0.05) (Figure 4a). Citrulline increased SOD activity in both N and IH conditions (Figure 4e). Concerning NO bioavailability, IH did not significantly impact nitrate levels (Figure 4f) but significantly decreased nitrite levels without any effect of citrulline (Figure 4g). IH did not significantly increase nitrotyrosine levels, but citrulline significantly decreased it only in the aorta of IH rats (5 ± 0.8 vs. 2.7 ± 0.6 µmol·g^−1^, in IH and IHCit groups, respectively, *p* < 0.05) (Figure 4h). Plasmatic nitrotyrosine levels were also significantly reduced in citrulline-supplemented IH rats (363.6 ± 10.3 vs. 269 ± 21.1 µmol·ml^−1^ in IH and IHCit groups, respectively, *p* < 0.0001) (Figure 4i).

### 3.4. Citrulline Prevented IH-Induced Increase in Superoxide Anion Content in Heart Tissue

**Figure 5:**
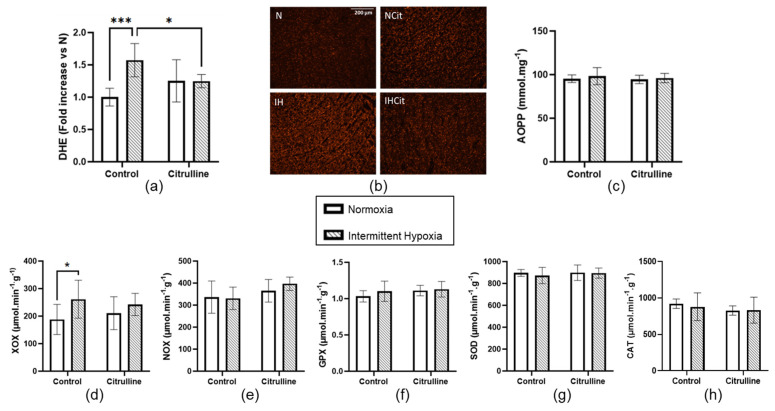
Citrulline decreased the intermittent hypoxia (IH)-induced increase in superoxide anion content in the heart. (**a**) DHE levels with (**b**) representative images (×100); (**c**) Concentration of advanced oxidation protein products (AOPP) in mmol·mg^−1^ of protein; (**d**) Activities of xanthine oxidase (XOX), (**e**) NADPH oxidase (NOX), (**f**) glutathione peroxidase (GPX), (**g**) superoxide dismutase (SOD) and (**h**) catalase (CAT) expressed in µmol·min^−1^·g^−1^ of protein (*n* = 5–6) of rats exposed to normoxia (N) or IH supplemented with 1 g·kg^−1^·d^−1^ citrulline (Cit) or not (Control). Values are represented as mean ± SD. Two-way ANOVA followed by Fisher’s LSD test. * *p* < 0.05, *** *p* < 0.001.

IH significantly increased DHE levels in cardiac tissue (1 ± 0.06 vs. 1.57 ± 0.11, in N and IH groups, respectively, *p* < 0.001), indicating an increase in superoxide anion content that is significantly abolished by citrulline supplementation (Figure 5a,b). IH did not impact AOPP (Figure 5c) nor NOX (Figure 5e), GPX (Figure 5f), SOD (Figure 5g), or CAT (Figure 5h) enzyme activities. Figure 5d shows that IH increased XOX enzyme activity significantly (187.9 ± 22.3 vs. 261.6 ± 30.8 µmol·min^−1^·g^−1^, in N and IH groups, respectively, *p* < 0.05) and that, in rats treated with citrulline, there is no difference between N and IH rats.

## 4. Discussion

This study shows that citrulline supplementation prevents IH-induced elevated BP and increased infarct size following an ischemia-reperfusion protocol. We further demonstrate that, only under IH, citrulline decreases aortic superoxide anion content, as well as aortic and plasmatic nitrotyrosine levels. In the heart, citrulline markedly reduces superoxide anion content and XOX enzyme activity, which have been significantly increased under IH.

### 4.1. Citrulline Did Not Impact the IH-Induced Decrease in Weight Loss and Hematocrit

Intermittent hypoxia slowed down the body weight gain and increased hematocrit, validating our model. These results are in accordance with previous studies demonstrating that rodents exposed to chronic IH tend to lose weight at the beginning of the exposure before gaining body weight after approximately 4 days [50]. In this study, we also demonstrated that food intake is decreased in hypoxic rats. Notably, the decrease in food intake was not fully responsible for the decrease in body weight gain since the normoxic rats submitted to a food restriction that adjusted to one of the hypoxic rats did not present the same body weight curve (Normoxic Pair-Fed group, Figure S1). In this study, the most important point was that citrulline was added to the food, suggesting that food intake and body weight have to be controlled daily. As evidenced by our results, these controls were rigorously performed since rats received nearly 1 g·kg^−1^ of citrulline daily in both groups, and it is important to mention that citrulline did not impact food intake (Figure 2b).

### 4.2. Citrulline Significantly Decreased Blood Pressure and Infarct Size under IH

As previously demonstrated by others and us, chronic IH increased BP and infarct size upon ischemia-reperfusion [18,51]. Notably, food restriction did not impact BP and infarct size (Figure S2a–d), meaning that IH, per se, induced an increase in both parameters. The current study demonstrates that citrulline prevents the occurrence of these deleterious cardiovascular consequences of IH. Our results are in line with previous studies demonstrating citrulline efficiency against hypertension in different animal models. The 0.25% of citrulline given in drinking water during 8 weeks decreased MBP in spontaneously hypertensive rats [35] and dahl salt-sensitive rats [52]. Here, we demonstrated that, in normotensive rats, citrulline prevents MBP increase induced by IH. Regarding the response to ischemia-reperfusion, Heidorn et al. demonstrated that citrulline improved cardiomyocyte contractility in vitro, as well as the post-ischemic recovery in isolated and perfused myocardium [37]. Furthermore, in db/db diabetic mice, citrulline combined with sebiapterin, a precursor of BH_4_, decreased infarct size, suggesting that citrulline can be protective against ischemia-reperfusion-induced cell death [38]. Here, we demonstrated that citrulline prevents infarct size increase induced by chronic IH. To our knowledge, this is the first study investigating citrulline efficiency in the context of chronic IH. The mechanisms underlying citrulline cardiovascular benefits have been poorly studied, and our study provides insights into citrulline capabilities in reducing oxidative stress.

### 4.3. Oxidative Stress in Aorta and Myocardium

Oxidative stress generated by IH is a key mechanism triggering BP elevation and endothelial dysfunction in OSA patients [53] and in rodents exposed to IH [27,48,54,55]. Superoxide anion production was reported to increase in the aorta and mesenteric arteries of mice exposed to 8 days of hypercapnic hypoxia [55] or to 14 days of IH (60 cycles/h; 21–5%) [54], while SOD activity decreased in rats exposed to 14 days of IH (60 cycles/h; 21–5%) [27]. Finally, NOX, XOX, and catalase activities were increased, whereas GPX activity was decreased in the aorta from rats exposed to 7 days of IH (10 cycles/h; 21–7%) [48]. These studies demonstrate different oxidative stress responses to IH among vascular beds that depend on the duration of total IH exposure as well as characteristics (number of cycles per hour, depth) of IH [56]. In recent meta-analyses performed on rodents, we demonstrated that the duration (acute exposure of few hours vs. chronic exposure of several days) and the depth of hypoxia (10% vs. 5%) impact the response to ischemia-reperfusion [57]. Since IH did not impact superoxide anion content or pro/anti-oxidant balance in the aorta, we cannot say that vascular oxidative stress is the main driver of an increase in BP under IH. However, IH tends to increase aortic and plasmatic nitrotyrosine levels, as previously demonstrated by the same stimulus in the aorta from hypoxic mice [54]. Citrulline supplementation significantly decreased the nitrotyrosine levels in the aorta and in plasma, solely in IH rats. Intimal eNOS-derived NO plays a major role in maintaining the vascular function, and eNOS uncoupling is associated with oxidative stress production [58,59,60]. However, the eNOS expression has been demonstrated to be increased after 3 days and drastically decreased after 8 weeks of IH, suggesting a compensatory followed by a deleterious mechanism associated with oxidative stress [61]. In our study, neither eNOS nor its inhibiting or activating phosphorylation on the serine1177 (S1177) and threonin495 (T495) residue were modified after 2 weeks of IH (Figure S3a,b). Accordingly, Siques et al. reported that chronic IH increased nitrotyrosine levels in pulmonary arteries without any modification in the eNOS expression [62]. Under IH, we observed a significant decrease in nitrite content, suggesting a partial decrease in NO availability. This could be explained by a decrease in eNOS activity triggered by ADMA production and/or by a decrease in arginine availability due to an increase in arginase activity as previously described under IH [30,63]. Regarding citrulline protective mechanisms, it was demonstrated to impact eNOS dynamics by increasing the total eNOS protein expression, S1177 phosphorylation, and NO production [52,64]. However, in the small pulmonary arteries of piglets, the vasorelaxation induced by citrulline was maintained in the presence of N(G)-Nitro-L-arginine methyl ester (L-NAME), this indicates that other factors than eNOS can explain the beneficial impact of citrulline on vascular tone [52]. This could explain why after 2 weeks of IH, citrulline decreased MBP without affecting the eNOS dynamics. The decrease in NO availability can also be explained by an increase in oxidative stress (i.e., O_2_^−^), leading to an increase in ONOO- and subsequent nitrosative stress. In our study, we reported that citrulline increased SOD activity in N and IH groups (Figure 4e). Citrulline especially decreased aortic and plasmatic nitrotyrosine levels only under IH and abolished the IH-decreased nitrites content (Figure 4g–i). This demonstrates that under IH, citrulline targets (1) oxidative stress and (2) the combination of oxidative stress with NO imbalance. 

Regarding the myocardium, we demonstrated that IH increased superoxide anion content and XOX activity. This is in agreement with previous studies demonstrating that chronic IH is a potent oxidative stress generator, particularly via XOX activation [65]. Previous studies demonstrated that myocardial superoxide anion production is associated with an increase in infarct size since anti-oxidative drugs decreasing superoxide anion or blocking its production also decreased cardiomyocyte death [18,27,28]. Our results showed that citrulline prevented the IH-increase in O_2_^−^ and XOX activity and also decreased infarct size without any modification in the eNOS activity and expression (Figure S3c,d). This is in agreement with other studies highlighting the potential anti-oxidant effects of citrulline: an increase in SOD activity and a decrease in lipid peroxidation in the heart tissue in a rat model of sepsis [66]; a blunt in H_2_O_2_^−^ induced ROS production in retinal epithelial cells while limiting lipid peroxidation and preventing cell death [67]; a renal decrease in oxidative-DNA damage without affecting the eNOS expression in spontaneously hypertensive rats [35]. This suggests that citrulline can preserve the heart against oxidative stress without any modification of the eNOS expression. This contributes to explain how, in the context of IH, citrulline decreased infarct size after an ischemia-reperfusion protocol. Finally, during ischemia-reperfusion, the decrease in ATP/ADP ratio slowed reactions requiring high energy levels. By thermodynamic action, previously described as a decrease in the energy activation levels of one or several ATP-consuming reactions [68,69], citrulline may protect against cardiomyocyte death.

Very interestingly, we observed that chronic IH altered both vascular and myocardial responses and that citrulline prevented these effects by exclusively reducing nitrosative stress in the aorta under IH and by significantly reducing superoxide anion content increased by IH in the heart. After 14 days of IH, we showed a photo (see graphical abstract) of a redox imbalance that deserves to be deeply investigated in further studies.

## 5. Study Limitation

In this study, we only used male rats, whereas OSA is also associated with cardiovascular disorders in women. However, women’s sensitivity and OSA-related diseases seem to differ depending on the hypoxic burden, reoxygenation, and hypoxia duration [70]. Likewise, females produced 36% more endogenous citrulline than males, which caused greater arginine production [71]. Then, citrulline supplementation increased plasmatic arginine more in men than women, which resulted in a decrease in DBP and in an increase in blood flow and vascular conductance in men while no change in women [72]. Even if these studies highlight a sexual dimorphism in arginine metabolism, they demonstrate that citrulline supplementation differentially impacts males and females. Considering also the differential impact of IH in males and females, there is no doubt that females should be considered in further studies [73].

## 6. Conclusions and Perspectives

To conclude, we demonstrated for the first time that citrulline protects against an IH-induced increase in MBP and infarct size but also decreases aortic and myocardial oxidative stress (see graphical abstract). Even if the specific role of citrulline on oxidative stress remains to be determined, the present study highlights that it can protect against the deleterious cardiovascular consequences of chronic IH, the major feature of OSA. Citrulline is a non-essential amino acid that can be safely administered in humans [39]. Citrulline may be used as the primary therapy after CPAP refusal or in combination with CPAP and/or lifestyle interventions. This remains to be established in clinical trials in OSA patients.

## Data Availability

Data is contained within the article and supplemental material.

## Supplementary Materials

The following supporting information can be downloaded at: https://www.mdpi.com/article/10.3390/antiox11122326/s1, Figure S1: Body weight gain of rats exposed to normoxia pair-fed (Npf) or intermittent hypoxia (IH); Figure S2: Intermittent hypoxia increase blood pressure and infarct size independently of food intake; Figure S3: Western blot analyses of endothelial nitric oxide synthase (eNOS) activity on the aorta and heart protein extracts.

## Appendix A

### Appendix A.1. Normoxia Pair-Fed (Npf) Group

Twenty Wistar male rats (6-week-old, 270–350 g, Janvier Labs, Le Genest-Saint-Isle, France) were housed at the animal care facility of the HP2 Laboratory under a 12:12 h light-dark cycle at 20 °C to 22 °C and allowed free access to standard food and water during one week of acclimatization. These rats, assigned to the Npf group, were submitted to N [14 d 8 h/d 21%O_2_] and given the same amount of food that the IH group had eaten (IH group rats were taken in protocol one week earlier than this group in order to measure daily food intake) to assess the impact of anorexia associated with IH. The first set of 14 animals was submitted to carotid catheterization and Langendorff methods, while the second set of six rats was used for tissue sampling.

### Appendix A.2. Western Blot

Proteins (30–50 µg) were separated by sodium dodecyl sulfate (SDS) polyacrylamide gels (8%–12%) and transferred to nitrocellulose membranes. Next, membranes were blocked with 5% BSA in Tris-buffered saline with Tween 20 (0.1%). Membranes were then incubated overnight at 4 °C with primary antibodies in Tris-buffered saline-Tween 20 (0.1%), 1% BSA. The following primary antibody was incubated: ADMA (1:1000, #13522 Cell Signaling), eNOS T495 (1:1000, #9574 Cell signaling), eNOS S1177 (1:1000, #9571 Cell Signaling), eNOS Total (1:1000, 610296 BD Transduction Laboratories), iNOS (1:1000, 610329 BD Transduction Laboratories), tubulin (1:1000, sc-5286, Santa Cruz Biotechnology). The following day, the membranes were incubated for 1 h at room temperature with the appropriate horseradish peroxidase–conjugated anti-IgG. Enhanced chemiluminescence (ECL) was performed with the western blot ECL substrate (Clarity; Bio-Rad) according to the manufacturer’s instructions, and images were acquired using the ChemiDoc-XRS-System (Bio–Rad). The relative amount of protein was quantified by densitometry (ImageJ) and expressed relative to tubulin. Phosphorylated proteins were expressed relative to total proteins.
