# Supplementary material for: L-Citrulline Supplementation Reduces Blood Pressure and Myocardial Infarct Size under Chronic Intermittent Hypoxia, a Major Feature of Sleep Apnea Syndrome
Source: Antioxidants (Basel). 2022 Nov 24;11(12):2326. doi: 10.3390/antiox11122326 (PMC9774116; doi:10.3390/antiox11122326)
Supplement: Supplementary file 1 [file antioxidants-11-02326-s001.zip › antioxidants-1991383-supplementary.pdf]

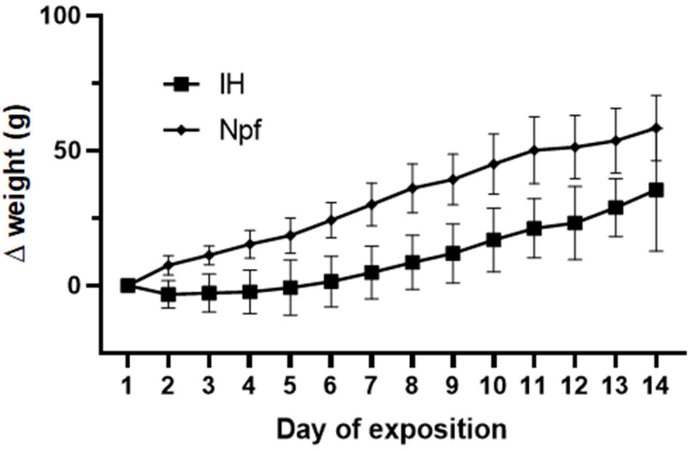

Figure S1. Body weight gain of rats exposed to normoxia pair-fed (Npf) or intermittent hypoxia (IH). Values are represented as mean ± SD.

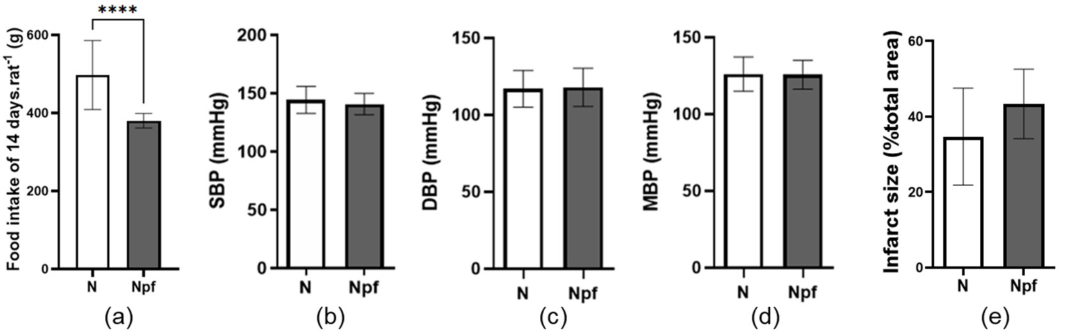

Figure S2. Intermittent hypoxia (IH) increased blood pressure and infarct size independent of food intake. (**a**) Food intake, (**b**) Systolic blood pressure (SBP), (**c**) diastolic blood pressure (DBP), (**d**) mean blood pressure (MBP) expressed in mmHg (n = 13–14), (**e**) infarct size expressed relative to total heart area (n = 13–14) from rats exposed to normoxia (N) or normoxia pair-fed (Npf).Values are represented as mean ± SD. *T-tests* are conducted. **** $p < 0.0001$.

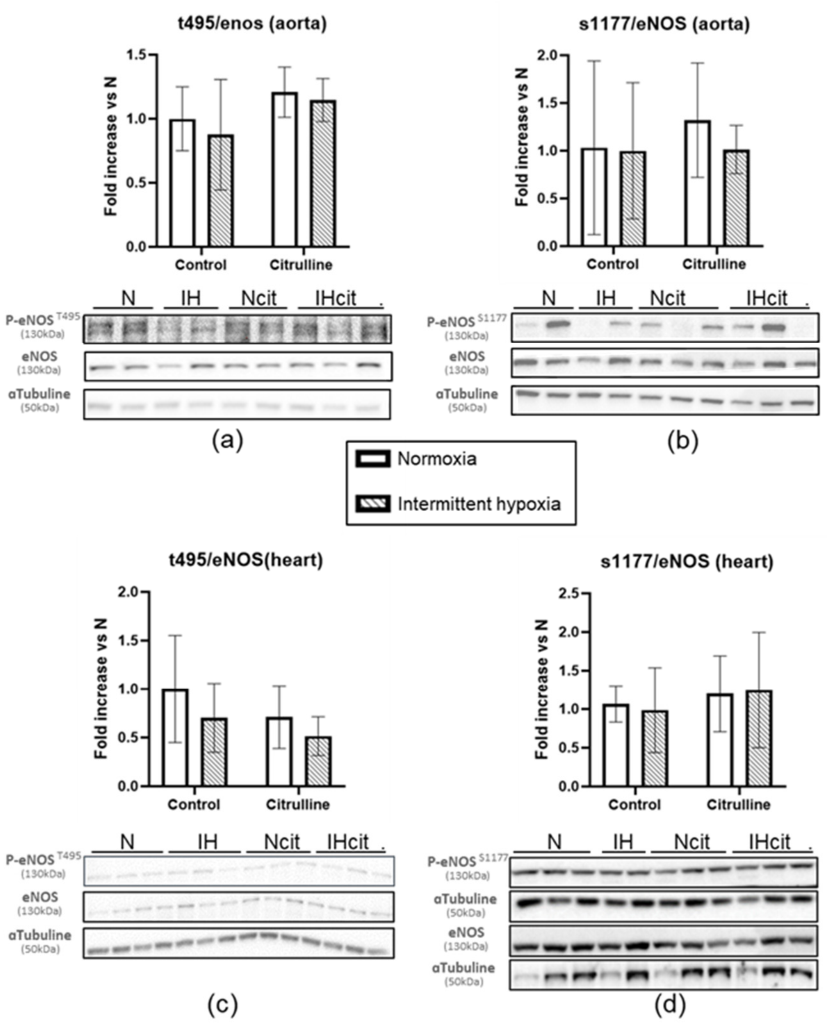

Figure S3. Western blot analyses of endothelial nitric oxide synthase (eNOS) activity on aorta and heart protein extracts. (**a**) eNOS phosphorylation at Threonine 495 (T495) and at (**b**) Serine 1177 (S1177) in aorta normalized to total eNOS and tubulin levels (n = 4–6); (**c**) eNOS phosphorylation at T495 and at (**d**) S1177 in heart normalized to total eNOS and tubulin levels (n = 5–6) of normoxia (N), or intermittent hypoxia (IH) exposed rats supplemented with 1 $g \cdot kg^{-1} \cdot d^{-1}$ citrulline (Cit) or not (Normal diet) (n = 4–6).Values are mean ± SD. ANOVA two-way, then Fisher's LSD tests were performed.
