# Supplementary figures and images for: L-Citrulline Supplementation Reduces Blood Pressure and Myocardial Infarct Size under Chronic Intermittent Hypoxia, a Major Feature of Sleep Apnea Syndrome
Source: Antioxidants (Basel). 2022 Nov 24;11(12):2326. doi: 10.3390/antiox11122326 (PMC9774116; doi:10.3390/antiox11122326)

## Slide 1
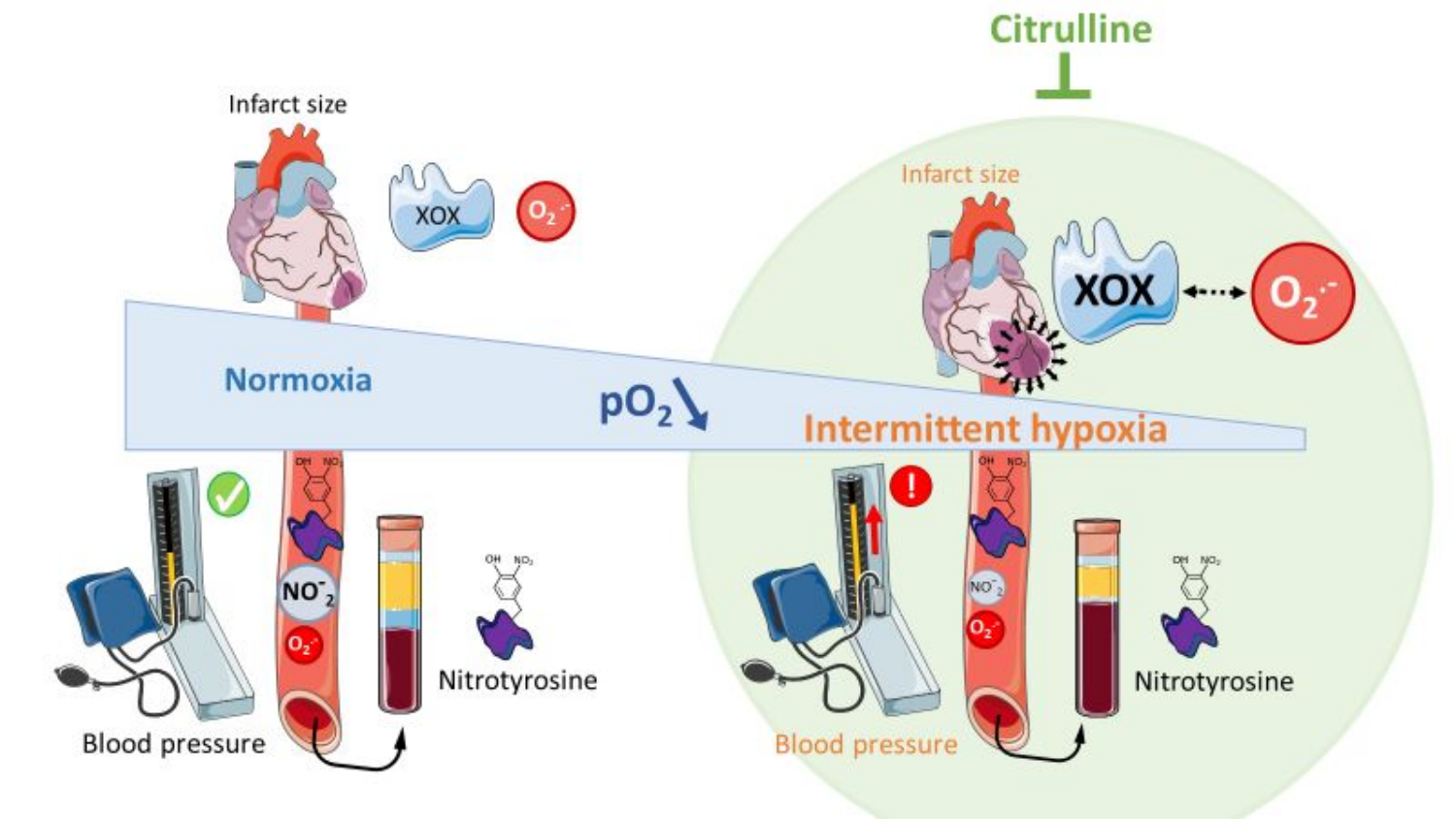

Supplement: Supplementary file 1 [file antioxidants-11-02326-s001.zip › antioxidants-1991383-supplementary.pptx]
